# Diagnosis and Management of Invasive Pleomorphic Lobular Carcinoma of the Breast: A Case Report and Comprehensive Review of Current Literature

**DOI:** 10.1002/cnr2.70285

**Published:** 2025-07-24

**Authors:** Xiaoyu Sun, Yanze Liu, Mei Wu, Jiaqi Liu

**Affiliations:** ^1^ Attending Physician, MM, Department of Pathology, Zibo Central Hospital Shandong Zibo China; ^2^ Resident Physician, MM, Department of Breast and Thyroid Surgery Zibo Central Hospital Shandong Zibo China; ^3^ Nurse‐In‐Charge, BS, Zibo Central Hospital Shandong Zibo China; ^4^ Attending Physician, PhD, Department of Breast and Thyroid Surgery, Zibo Central Hospital Shandong Zibo China

**Keywords:** breast cancer, chemotherapy, imaging challenges, invasive pleomorphic lobular carcinoma, lymph node metastasis, modified radical mastectomy

## Abstract

**Introduction:**

Invasive pleomorphic lobular carcinoma (IPLC) is a rare and aggressive variant of invasive lobular breast cancer, characterized by its distinct histological features and potential for metastatic spread. This case report aims to highlight the diagnostic challenges of IPLC, particularly regarding its imaging and pathological characteristics, and to review the latest treatment protocols.

**Case Presentation:**

A 43‐year‐old female presented with a painless right breast mass that had persisted for 4 months. The breast ultrasound and mammography did not provide an accurate assessment, whereas MRI indicated multiple abnormal signals throughout the right breast, classified as BI‐RADS 4c. Surgical intervention and subsequent pathological examination confirmed the diagnosis of multifocal invasive pleomorphic lobular carcinoma with lymph node metastasis.

**Conclusion:**

IPLC presents diagnostic challenges due to its rare and aggressive nature, requiring a high degree of clinical suspicion. This case underscores the importance of comprehensive imaging and pathological assessment for the accurate diagnosis and treatment of IPLC. The standard treatment involves modified radical mastectomy, lymph node dissection, and postoperative chemotherapy.

**Abbreviations**
AR: androgen receptor
BC: breast cancer
CAAs: cancer‐associated adipocytes
CAFs: cancer‐associated fibroblasts
CDFI: color doppler flow imaging
ctDNA: circulating tumor DNA
ER: estrogen receptor
HER2: human epidermal growth factor receptor 2
HSL: hormone‐sensitive lipase
IHC: immunohistochemical
ILC: invasive lobular carcinoma
IPLC: invasive pleomorphic lobular carcinoma
MRI: magnetic resonance imaging
NACT: neoadjuvant therapy
OS: overall survival
PFS: progression‐free survival
PLCIS: pleomorphic lobular carcinoma in situ
PR: progesterone receptor
TAMs: tumor‐associated macrophages
TILs: tumor‐infiltrating lymphocytes
TME: tumor microenvironment
TNBC: triple‐negative breast cancer

## 1 Introduction

Invasive pleomorphic lobular carcinoma (IPLC) is a rare and aggressive variant of invasive lobular carcinoma (ILC), accounting for less than 1% of all breast cancer (BC) [1]. Characterized by its distinct histological features—including marked nuclear pleomorphism, loss of E‐cadherin expression, and frequent apocrine differentiation—IPLC exhibits a higher propensity for metastatic spread and poorer prognosis compared to classical ILC. Unlike the well‐differentiated cells of classic lobular carcinoma, IPLC displays significant cytological atypia, high mitotic activity, and a Ki‐67 proliferation index often exceeding 20% [2], reflecting its aggressive biological behavior [3].

The diagnostic challenges of IPLC stem from its insidious clinical presentation and imaging limitations [4]. Clinically, IPLC may present as a subtle, ill‐defined breast mass or, in some cases, manifest primarily through axillary lymph node metastasis, mimicking occult BC. Radiologically, conventional imaging modalities such as mammography and ultrasound frequently underestimate the extent of disease due to the tumor's diffuse growth pattern and lack of desmoplastic reaction [5]. Magnetic resonance imaging (MRI), while more sensitive in detecting multifocal lesions, often reveals non‐specific enhancement patterns. Histopathology is necessitated anyway, mass enhancement or non‐mass.

Current treatment paradigms for IPLC align with guidelines for high‐risk BC, emphasizing multimodal therapy [6]. Mastectomy with axillary lymph node dissection remains the cornerstone of surgical management, followed by adjuvant chemotherapy, targeted therapy (e.g., HER2‐directed agents if HER2 positive), and radiation [7]. However, the absence of hormone receptor expression in a subset of IPLC cases limits the utility of endocrine therapy, underscoring the need for tailored therapeutic strategies. Despite advances in molecular profiling, the rarity of IPLC has hindered large‐scale studies, leaving critical gaps in understanding its genomic drivers and optimal management.

This report aims to explore the diagnosis, treatment, and prognostic evaluation of IPLC of the breast through the clinical, imaging, and pathological data of a single patient.

## 2 Case Presentation

A 43‐year‐old female patient presented to Zibo Central Hospital on November 18, 2024, with a painless mass in the right breast that had persisted for 4 months. The patient had no significant medical history or family history of BC.

Physical Examination by Specialist: A mass measuring approximately 2.0 × 1.0 cm was palpable in the right breast, located at the 12 o'clock position, 5 cm from the nipple. The mass was firm, with unclear borders, a smooth surface, and mobile. There was no adhesion to the skin or pectoral muscle, and no pain upon palpation. No nipple discharge was observed. No enlarged lymph nodes were detected in the axillary or supraclavicular regions.

**FIGURE 1:**
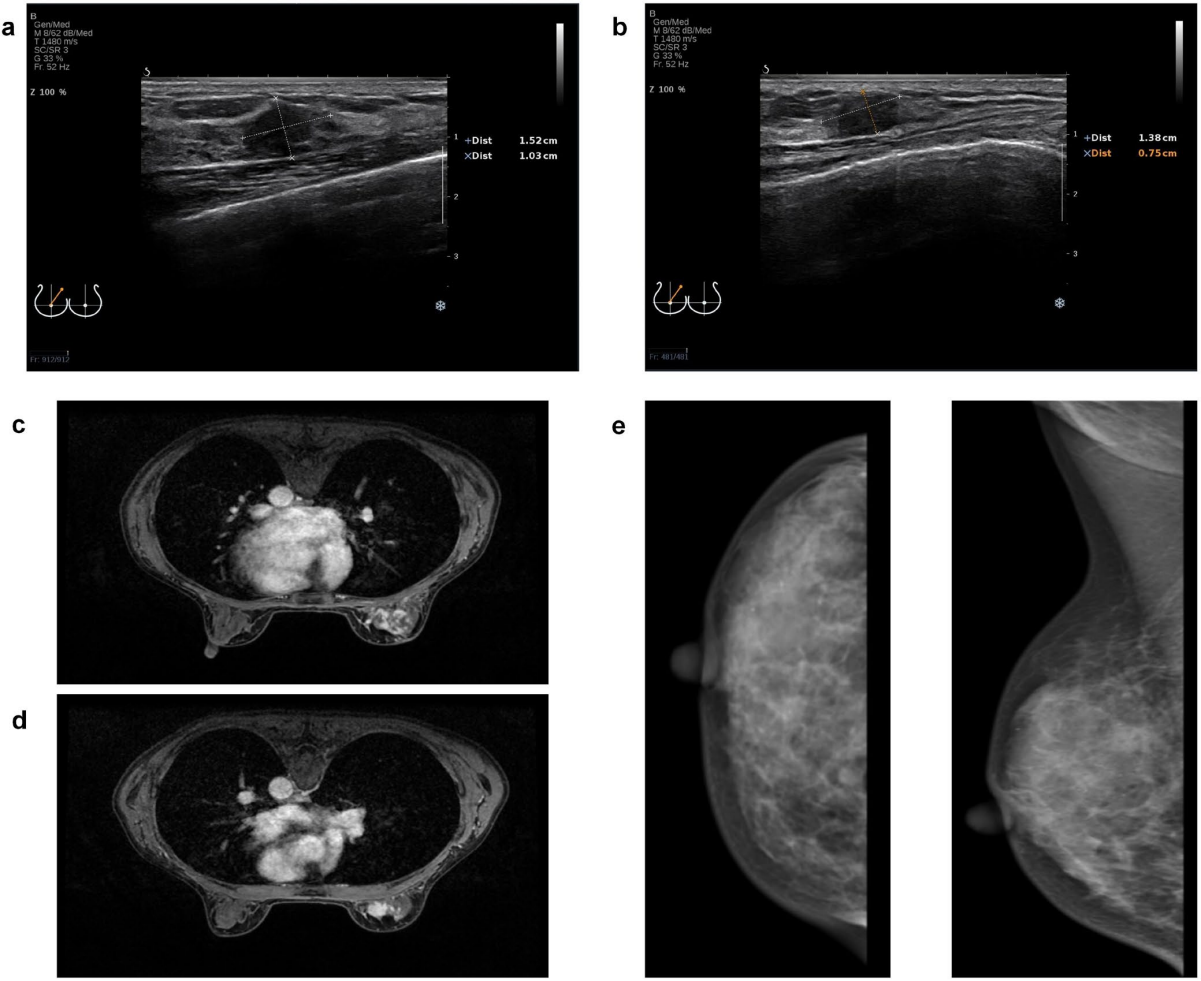
Diagnostic imaging findings. (a, b) Ultrasound images of the affected breast. (c, d) Contrast‐enhanced breast magnetic resonance imaging (MRI). (e) Mammographic images of the affected breast (craniocaudal and mediolateral oblique views).

Ultrasound Examination: The right breast showed multiple low‐echo nodules, with the largest measuring approximately 15 × 10 mm (Figure 1a) and 14 × 8 mm (Figure 1b), respectively. The nodules had unclear borders, irregular shapes, and heterogeneous internal echoes. Color Doppler Flow Imaging (CDFI) showed blood flow within the nodules. No enlarged lymph nodes were detected in the bilateral axillary regions.

Mammography: The right breast showed a high‐density mass in the upper inner quadrant, measuring approximately 12 × 10 mm, with blurred margins and irregular shape (Figure 1e). Multiple scattered heterogeneous calcifications were also observed. No enlarged lymph nodes were detected in the bilateral axillary regions.

Breast MRI: The right breast showed multiple abnormal signals, with the largest lesion located in the upper inner quadrant, measuring approximately 1.9 × 1.1 × 2.0 cm (Figure 1c,d). The lesions had irregular margins, heterogeneous signals, and showed heterogeneous enhancement on contrast‐enhanced scans. Enlarged lymph nodes were observed in the right axilla, with the largest measuring approximately 1.3 × 0.7 cm (Figure 1c,d).

Surgical Intervention: On November 19, 2024, the patient underwent a modified radical mastectomy. Intraoperative pathological examination of the sentinel lymph nodes revealed metastatic carcinoma in 1 out of 2 lymph nodes. Further axillary lymph node dissection was performed.

**FIGURE 2:**
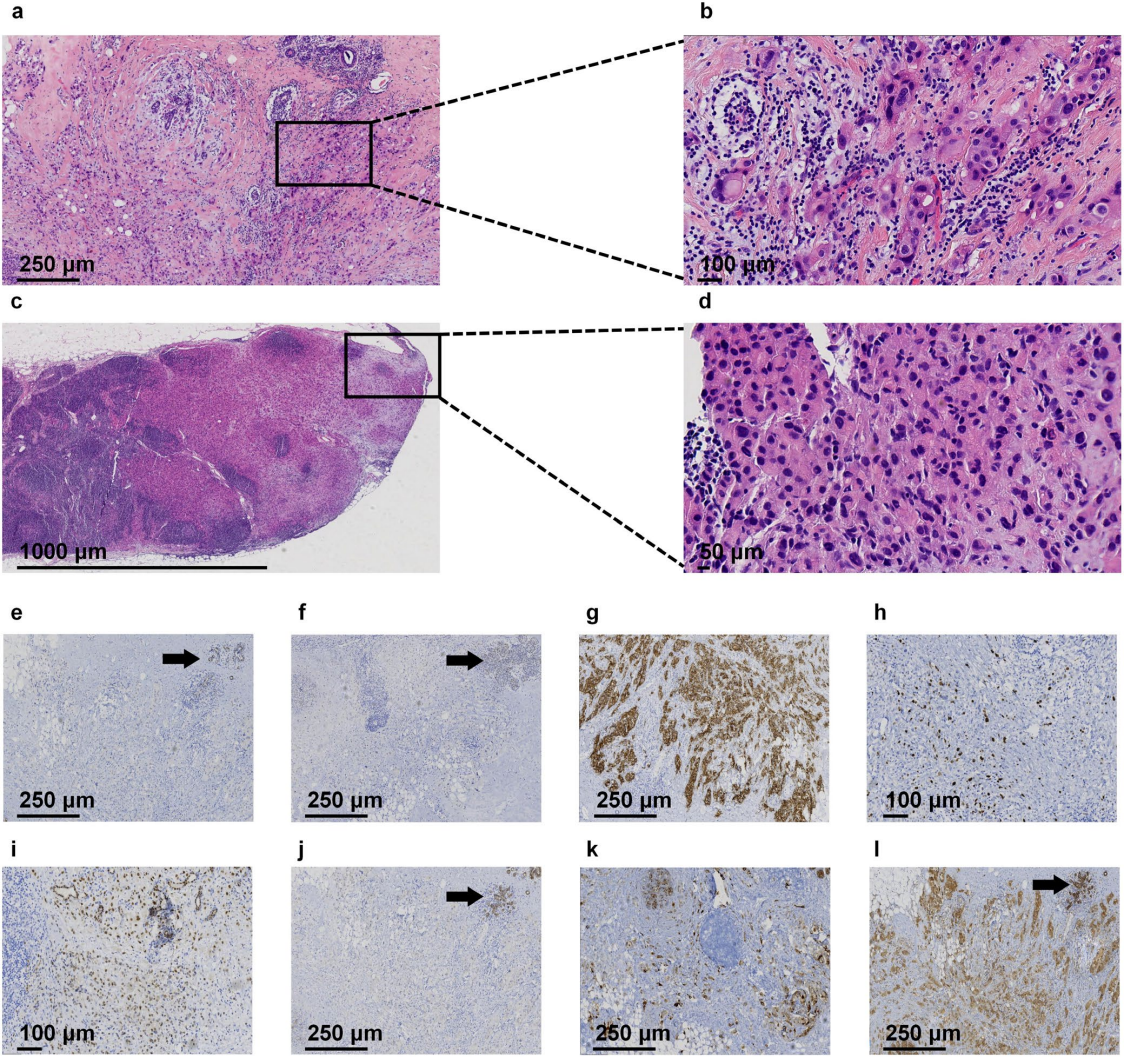
Histopathological and immunohistochemical characterization of IPLC. (a) H&E staining (×100 magnification; scale bar: 250 μm). The tumor exhibits an invasive growth pattern with the destruction of adjacent normal breast lobules. (b) H&E staining (×200 magnification; scale bar: 100 μm). Higher magnification of the boxed area in a reveals pleomorphic tumor cells with eosinophilic cytoplasm and nuclear atypia. (c) Lymph node metastasis (H&E, ×40 magnification; scale bar: 1000 μm). Metastatic tumor nests are visible within the lymph node parenchyma. (d) Lymph node metastasis (H&E, ×400 magnification; scale bar: 50 μm). Higher magnification of the boxed area in c shows tumor cells morphologically consistent with the primary breast lesion. (e) Immunohistochemistry (IHC) of ER (×100 magnification; scale bar: 250 μm). Tumor cells are negative, while internal control (ductal epithelium) shows positive nuclear staining (arrow). (f) IHC of PR (×100 magnification; scale bar: 250 μm). Tumor cells are negative, with positive nuclear staining in adjacent normal ducts (arrow). (g) IHC of HER2 (×100 magnification; scale bar: 250 μm). Tumor cells exhibit strong diffuse membranous staining (3+; HER2‐positive). (h) IHC of Ki‐67 (×200 magnification; scale bar: 100 μm). Tumor nuclei show high proliferative activity (Ki‐67 index ≥ 30%). (i) IHC of AR (×200 magnification; scale bar: 100 μm). Tumor cells display diffuse nuclear positivity for androgen receptor. (j) IHC of E‐cadherin (×100 magnification; scale bar: 250 μm). Tumor cells are negative, with preserved membranous staining in normal ductal epithelium (arrow). (k) IHC of GCDFP‐15 (×100 magnification; scale bar: 250 μm). Tumor cells show diffuse cytoplasmic positivity, supporting apocrine differentiation. (l) IHC of p120‐catenin (×100 magnification; scale bar: 250 μm). Tumor cells exhibit cytoplasmic accumulation (loss of membranous staining), while normal ducts retain membranous p120 expression (arrow).

Postoperative Pathology: The right breast showed diffuse multifocal invasive pleomorphic lobular carcinoma, Grade III, with partial apocrine differentiation (70% of tumor area), coexisting with pleomorphic lobular carcinoma in situ (30% of tumor area) and stromal lymphocyte infiltration (tumor‐infiltrating lymphocytes [TILs] approximately 5%). The largest tumor measured 4 × 3.5 × 1.2 cm, with additional foci measuring 3.5 × 2 × 0.5 cm. Histopathological examination confirmed tumor invasion into the subnipple tissue, stromal lymphovascular spaces, and perineural bundles. Surgical margins (peripheral and deep) were free of carcinoma. Lymph Node Metastasis: Right sentinel lymph nodes: 1 out of 2 nodes positive for metastatic carcinoma. Right axillary lymph nodes: 2 out of 13 nodes positive for metastatic carcinoma. Immunohistochemistry: E‐cadherin: Positive (+); p120: Cytoplasmic (+); CK5/6, P63: Myoepithelial (+); GCDFP‐15: Positive (+); ER: Negative (−); PR: Negative (−); AR: Moderately positive (++, 60% of cells); HER2 (4B5): Strongly positive (3+); p53: Positive (mutant pattern); Ki‐67: High proliferation index (60% of cells) (Figure 2).

The specimens were fixed in 3.7% neutral‐buffered formalin, processed according to standardized BC specimen handling protocols, and sectioned for routine hematoxylin and eosin (H&E) staining. Whole‐slide images were digitized using a Jiangfeng digital pathology slide scanner. Immunohistochemical (IHC) antibodies, including p120, E‐cadherin, human epidermal growth factor receptor 2 (HER2), estrogen receptor (ER), progesterone receptor (PR), androgen receptor (AR), GCDFP‐15, and Ki‐67, were procured from Fuzhou Maixin Biotechnology Development Co. Ltd. All slides were independently reviewed by two board‐certified pathologists with associate chief physician qualifications.

Postoperative Treatment: The patient was administered the TCbHP chemotherapy regimen for 6 cycles, with 1 cycle every 21 days. The regimen included albumin‐bound paclitaxel, carboplatin, trastuzumab, and pertuzumab. Following chemotherapy, the patient underwent 25 sessions of radiation therapy. Trastuzumab and pertuzumab were continued for 1 year. After 1 year, neratinib was introduced as an adjuvant targeted therapy.

Adverse/unanticipated event: The patient experienced grade 1 nausea and vomiting (Common Terminology Criteria for Adverse Events [CTCAE] version 5.0) following chemotherapy. Symptomatic management with ondansetron (8 mg intravenous infusion) and fosaprepitant (150 mg intravenous infusion) resulted in complete resolution of emesis within 24 h.

**FIGURE 3:**
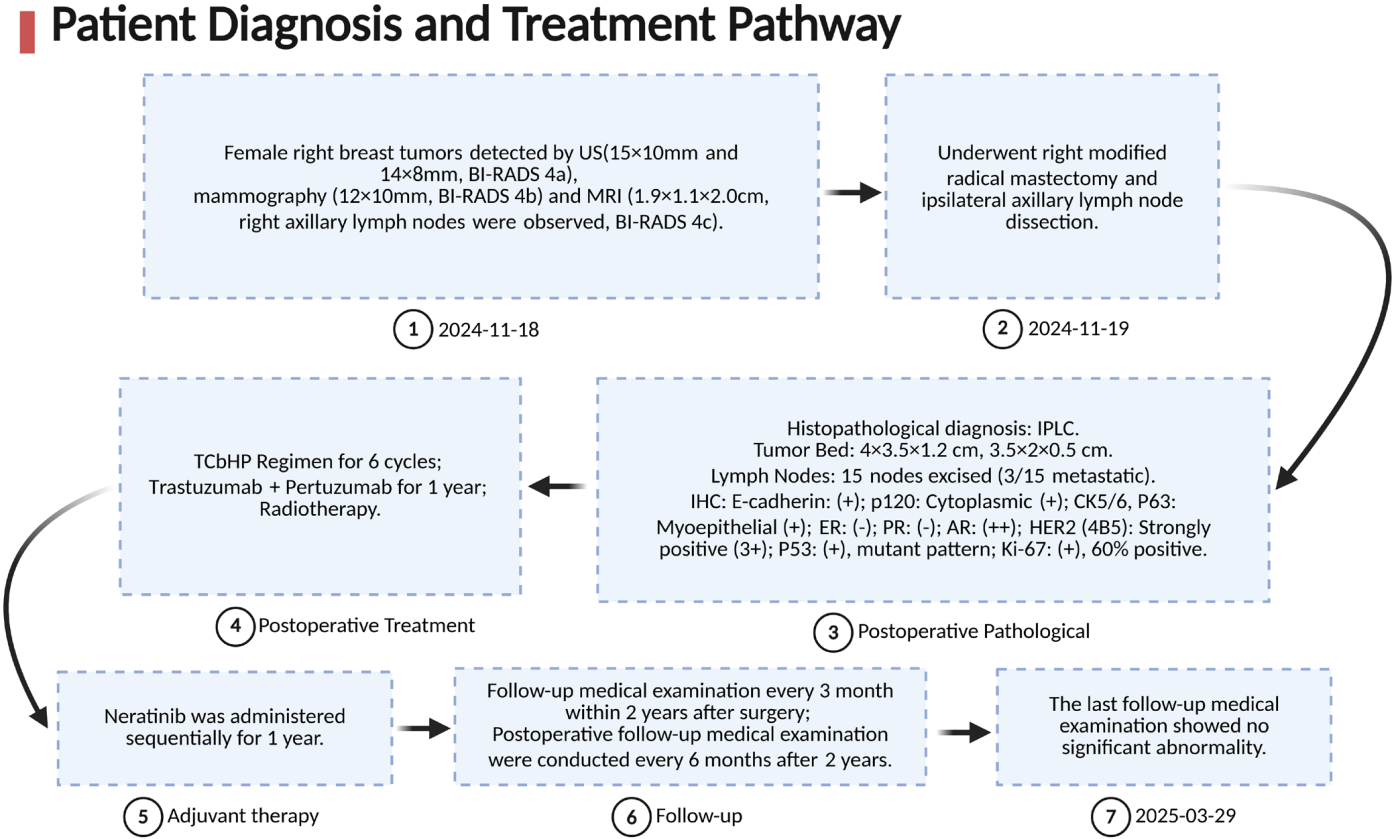
IPLC patient diagnosis and treatment pathway. AR, androgen receptor; ER, estrogen receptor; HER2, human epidermal growth factor receptor 2; IHC, immunohistochemistry; IPLC, invasive pleomorphic lobular carcinoma; MRI, magnetic resonance imaging; PR, progesterone receptor; TCbHP, T, Paclitaxel; Cb, Carboplatin; H, Trastuzumab; P, Pertuzumab.

Concluding remarks: The patient's diagnosis and treatment process is shown in Figure 3. The National Comprehensive Cancer Network (NCCN) guidelines recommend neoadjuvant therapy (NACT) as an effective strategy for high‐risk breast cancer subtypes (e.g., triple‐negative or HER2‐positive) [7]. However, in this case, the inability to obtain a definitive pathological diagnosis preoperatively due to inadequate diagnostic workup (patient declined core needle biopsy) precluded NACT and potential eligibility for breast‐conserving surgery (BCS).

## 3 Discussion

IPLC represents a rare and aggressive subtype of breast cancer, accounting for approximately 15% of ILC [8]. IPLC shares many features with traditional ILC but exhibits a higher degree of pleomorphism, multifocality, and a more aggressive clinical course. Our summary of prior case reports on IPLC (Table 1) demonstrates that the current case similarly highlights the diagnostic and therapeutic challenges associated with IPLC, particularly its multifocal presentation, HER2‐positive status, and high proliferative activity. The patient's clinical, imaging, and histopathological findings align with established IPLC characteristics while highlighting unique features that warrant deeper exploration.

**TABLE 1:** Published studies on IPLC case series.

Year		2020	2020	2020		2021		2022	2024	2024	2024
References		Taniguchi K et al. [9]	Nelson B et al. [10]	Burky M J et al. [11]		Chen X et al. [12]		Aljhdali H et al. [13]	Rogges E et al. [14]	Jinadasa M et al. [15]	Shen F et al. [16]
*n*		31	1	1		1		1	1	1	1
Age		40–86	57	70		42		86	46	28	50
Presentation		NR	Palpable mass	Palpable mass		Palpable mass		Palpable mass	Palpable mass	Palpable mass	Palpable mass
Size		NR	35 mm (Mammogram)	15 mm (Ultrasound)	6 mm (Ultrasound)	20 mm (Ultrasound)	20 mm	49 mm (Mammogram)	40 mm	80 mm (Mammogram)	NR
Primary BC		IPLC	IPLC	IPLC	ILC	IPLC	IPLC	IPLC	IPLC	IPLC	ILC
Metastasis		ALNM (*n* = 14); DM (*n* = 14)	ALNM: 1/18	ALNM: 0/3		DM (Liver, vertebral body, appendage of the whole spine, sternum)		ALNM: 2/2	ALNM	Neg	ALNM: 27/28; DM (small intestinal)
Pathological features	ER	Pos (*n* = 12)	−	+++ (90%)	+++ (95%)	−	+++ (65%)	+++	+ (100%)	−	++ ~ +++ (90%)
	PR	Pos (*n* = 4)	−	+++ (80%)	+++ (95%)	−	+++ (80%)	+++	+ (100%)	−	+++ (95%)
	HER2	Pos (*n* = 8)	3+	2+ (FISH: Pos)	1+	2+ (FISH: Pos)	3+	−	2+ (FISH: Neg)	−	−
	Ki‐67	< 30% (*n* = 22) ≥ 30% (*n* = 6)	NR	NR	NR	+ (30%)	+ (8%)	NR	+ (70%)	+ (50%)	+ (30%)
	E‐cadherin	NR	−	−	+	−	−	−	−	−	+
	GCDFP15	Pos (*n* = 19)	NR	NR	NR	NR	NR	NR	NR	NR	+
	AR	Pos (*n* = 23)	NR	NR	NR	NR	NR	NR	NR	NR	NR
	P120	NR	NR	NR	NR	+ (cytoplasmic)	NR	NR	NR	NR	NR
	CK5/6	NR	NR	NR	NR	NR	+ (myoepithelial)	NR	NR	NR	NR
	GATA‐3	NR	NR	NR	NR	NR	NR	NR	NR	+ (myoepithelial)	NR
	P53	NR	NR	NR	NR	NR	NR	NR	NR	+ (myoepithelial)	NR
Treatment		NR	TCHP for 6 cycles → Surgery → Tz for 14 cycles	Surgery → TCH for 6 cycles →Tz → ET		PH for 9 cycles → Pyrotinib + CAP		Letrozole for 1 month → Surgery → RT → Letrozole for 5 years	Surgery → Chemo → RT	NACT → Surgery → RT	Surgery →EC‐T for 8 cycles → RT → Anastrozole + Gos
Outcome		5 Died (20.7‐month follow‐up)	NR	No recurrence (8‐month follow‐up)		Stable disease (12‐month follow‐up)		No recurrence (9‐month follow‐up)	No recurrence (12‐month follow‐up)	NR	DM (63‐month follow‐up)

Abbreviations: ALNM, axillary lymph node metastasis; AR, androgen receptor; BC, breast cancer; CAP, capecitabine; Chemo, chemotherapy; DM, distant metastasis; EC‐T, E, epirubicin; C, cyclophosphamide; T, paclitaxel, ER, estrogen receptor; ET, endocrine therapy; Gos, goserelin; HER2, human epidermal growth factor receptor 2; ILC, invasive lobular breast carcinoma; IPLC, invasive pleomorphic lobular carcinoma; NACT, neoadjuvant chemotherapy; NR, not reported; PH, P, paclitaxel; H, trastuzumab; PR, progesterone receptor; RT, radiation therapy; TCH, T, docetaxel; C, carboplatin; H, trastuzumab; TCHP, T, docetaxel; C, carboplatin; H, trastuzumab; P, pertuzumab; Tz, Trastuzumab.

### 3.1 Imaging Challenges and Multifocality

The multifocal nature of IPLC, as observed in this patient's MRI, with multiple irregular masses throughout the right breast, underscores the limitations of conventional imaging modalities. However, mammography and ultrasonography initially classified the lesion as BI‐RADS 4a/4b, failing to delineate the full extent of disease. IPLC often results in subtle and poorly defined lesions that are difficult to detect on routine imaging. MRI, however, revealed diffuse involvement with several masses scattered throughout the breast (BI‐RADS 4c), consistent with studies demonstrating MRI's superior sensitivity (92%–98%) in detecting multifocal lesions compared to mammography (60%–75%) [4]. This aligns with the 2023 European Society of Breast Imaging (ESUR) guidelines, which recommend preoperative MRI for suspected lobular carcinomas due to their infiltrative growth patterns [17]. The discordance between imaging modalities in this case emphasizes the necessity of MRI for accurate staging and surgical planning in IPLC, particularly when multifocality is suspected.

### 3.2 Pathological and Molecular Heterogeneity

Histologically, IPLC retains many hallmark features of traditional lobular carcinoma but exhibits a greater degree of pleomorphism. The tumor cells show marked variation in size and shape, with a high nuclear‐to‐cytoplasmic ratio and frequent mitotic activity. The infiltrative, discohesive growth pattern, characteristic of lobular carcinoma, is also present, contributing to the difficulty in diagnosing this subtype [18]. The nuclei are irregular, with prominent nucleoli and frequent mitotic figures, which are typical of aggressive BC [8]. The tumor often has significant glandular‐like elements, further complicating its diagnosis. Immunohistochemical findings are critical for diagnosing IPLC. The tumor cells typically express p120 protein and show E‐Cadherin negativity or weak positivity, which are hallmarks of lobular carcinoma [8]. The ER expression rate is lower in IPLC than in classical lobular carcinoma, with approximately 30%–80% of cases showing HER2 overexpression [19], a distinct feature of IPLC. Furthermore, 5%–10% of cases may be positive for ER, PR, and HER2, which is consistent with the more aggressive behavior observed in IPLC. P53 is frequently positive in 20%–60% of IPLC cases, highlighting its role in tumor progression. This combination of histological features and immunohistochemical profiles underlines the unique biological behavior of IPLC and its distinction from other BC subtypes, emphasizing the need for accurate diagnosis and personalized treatment strategies.

In this case, immunohistochemical analysis confirmed HER2 (3+), a feature not typically associated with ILC, as HER2 amplification is found in only 5%–10% of classic ILC cases [20]. The HER2‐positive status is particularly significant as it correlates with increased aggressivity and a worse prognosis, with a research showing an increased recurrence risk for HER2‐positive IPLC [2]. Additionally, the high Ki‐67 index of 60% further underscores the tumor's proliferative vigor, which is a known predictor of poor outcomes, especially in hormone receptor‐negative BC [21].

The absence of ER/PR expression in this case, along with the presence of AR positivity (60%), adds complexity to the hormonal profile. AR positivity in triple‐negative breast cancer (TNBC) has been linked to potential responsiveness to anti‐androgen therapies, though its role in HER2‐positive IPLC remains largely unexplored. Notably, the tumor also demonstrated pleomorphic lobular carcinoma in situ (PLCIS), which is increasingly recognized as a precursor lesion to IPLC [22]. PLCIS is characterized by nuclear pleomorphism and loss of E‐cadherin expression, as seen in this case (E‐cadherin with cytoplasmic p120 staining, typical of lobular phenotype). The coexistence of PLCIS and IPLC supports the “lobular neoplastic spectrum” model, where PLCIS may progress to invasive disease [23, 24].

Furthermore, apocrine differentiation (GCDFP15+) observed here is understudied in IPLC. The apocrine differentiation, marked by GCDFP15 positivity, may reflect a distinct molecular pathway, potentially linked to metabolic reprogramming, as suggested by recent proteomic studies [25].

In recent years, the tumor microenvironment (TME) has garnered increasing attention for its diagnostic value in oncology [19]. Blood‐based biomarkers derived from the TME are frequently studied as candidate targets for tumor detection. Tille, J C et al. and Desmedt, C et al. reported that tumor‐infiltrating lymphocytes (TILs) are associated with younger age, larger tumor size, lymph node involvement, HER2 amplification, multinucleation, and prominent nucleoli [26, 27]. These findings provide critical insights for clinical diagnosis. Tumor‐associated macrophages (TAMs) are broadly categorized into M1 (anti‐tumor) and M2 (pro‐tumorigenic) subtypes based on their functional roles [28]. Notably, ILC exhibits a higher concentration of M2‐like macrophages [29, 30, 31], and quantifying M2‐like macrophage infiltration may enhance diagnostic guidance for clinicians. In classic ILC, cancer‐associated fibroblasts (CAFs) expressing alpha‐^+^SMA dominate the TME. In contrast, IPLC demonstrates a significant increase in S100‐expressing CAFs adjacent to tumor cells. This discovery underscores the heterogeneity of the TME across ILC subtypes and offers novel perspectives for diagnosing IPLC. Furthermore, cancer‐associated adipocytes (CAAs) in ILC exhibit elevated expression of hormone‐sensitive lipase (HSL) and fatty acid‐binding protein 4 (FABP4), alongside reduced perilipin A levels. These distinct molecular signatures may serve as auxiliary diagnostic markers for IPLC.

### 3.3 Therapeutic Implications of HER2 and AR Positivity

The patient's ER‐negative, PR‐negative, and HER2‐positive status guided the selection of the TCbHP regimen, consisting of trastuzumab, pertuzumab, carboplatin, and nab‐paclitaxel. This combination is a well‐established treatment for HER2‐positive breast cancers, particularly in metastatic settings, as demonstrated by the CLEOPATRA trial, which showed improved progression‐free survival (PFS) and overall survival (OS) in HER2‐positive metastatic breast cancer. In early‐stage disease, the APHINITY trial demonstrated that dual HER2 blockade significantly reduces recurrence risk by 19% in node‐positive patients [32]. Despite the proven efficacy of HER2‐targeted therapies in HER2‐positive BC, data specific to IPLC are scarce, and further studies are required to establish their effectiveness in this unique subtype.

Additionally, the ExteNET trial demonstrated the efficacy of neratinib, an irreversible pan‐HER inhibitor, in reducing the risk of recurrence in HER2‐positive patients following chemotherapy and HER2 blockade [33]. The addition of neratinib in this patient's treatment plan, post‐chemotherapy, could further reduce the risk of recurrence, particularly in high‐risk, HER2‐positive patients like this one.

According to the NCCN guidelines, AR‐targeted therapy is recommended solely as a personalized therapeutic option for advanced breast cancer patients with AR‐positive tumors [7]. Currently, research on AR in IPLC remains limited. Emerging studies indicate that AR positivity is significantly higher in TNBC‐invasive lobular carcinoma compared to TNBC‐invasive ductal carcinoma [34]. Furthermore, HER2‐positive breast cancer patients exhibit a positive correlation between AR pathway activity and AR expression status [35], suggesting potential benefits from AR antagonists in HER2+/AR+ subtypes. AR positivity is frequently associated with molecular alterations in PI3K, ERBB2, and ESRRA [34]. Notably, IPLC also demonstrates prevalent PI3K and ERBB2 mutations [8]. Targeting these mutations (PI3K and ERBB2) in conjunction with anti‐AR therapy may pave the way for novel combinatorial targeted therapeutic strategies.

### 3.4 Prognostic Considerations and Surveillance

Postoperative surveillance was designed in accordance with the NCCN Clinical Practice Guidelines for Breast Cancer and referenced follow‐up protocols for other breast cancer subtypes. The implemented follow‐up regimen included:

Frequency: Clinical evaluations every 3 months for the first 2 years post‐surgery, transitioning to every 6 months thereafter.

Investigations: Breast and axillary lymph node ultrasound; Abdominal and pelvic (uterus/adnexa) ultrasound; Chest CT; Routine blood biochemistry and tumor marker panels.

This patient's axillary lymph node metastasis (3/15 nodes) and extensive local invasion (including nipple involvement and perineural infiltration) portend a guarded prognosis. A recent study in patients with IPLC reported that positive lymph nodes were strongly associated with poor prognosis in IPLC patients [36]. The high Ki‐67 index and HER2 positivity in this patient amplify the risk of recurrence, emphasizing the need for vigilant surveillance.

Recent studies have demonstrated that circulating tumor DNA (ctDNA), as a pivotal biomarker in liquid biopsy, exhibits significant potential for monitoring early recurrence in breast cancer [37]. As demonstrated in the c‐TRAK TN trial, which showed that ctDNA‐guided interventions could provide early insight into disease recurrence [38]. IPLC is frequently associated with CDH1 gene abnormalities. Future studies should investigate the detection sensitivity of IPLC‐specific mutations (e.g., CDH1 alterations) in ctDNA to improve the combined utility of ctDNA and imaging surveillance for IPLC monitoring.

Additionally, TME characteristics provide a strategic direction for the development of novel biomarkers. TILs are closely associated with immune checkpoint blockade (e.g., PD‐L1 inhibition) and targeted therapies [19]. Tille, J C et al. and Desmedt, C et al. demonstrated that ILC harbors an immune‐enriched microenvironment that influences tumor behavior [26, 27], suggesting potential benefits of immunotherapy for IPLC. ILC is characterized by a high density of M2‐like macrophages [29, 30, 31], which may impair T‐cell infiltration and tumor cell engagement. Therefore, remodeling the M2‐dominant TME in IPLC may enhance endogenous T‐cell‐mediated antitumor activity. Emerging studies indicate that specific CAFs markers, such as CAF‐S1 (CD29^Medium^ FAP^Hi^ FSP1^Low‐Hi^ αSMA^Hi^ PDGFRβ^Medium‐Hi^ CAV1^Low^) [39] and FAP^Positive^ cells [19], exhibit immunosuppressive properties. Targeting CAFs within the IPLC TME represents a promising therapeutic strategy. CAAs mediate fatty acid release and regulate inflammatory mediators and protumorigenic molecules, thereby reprogramming the TME to facilitate tumor progression. Therapeutic interventions targeting CAAs may offer clinical benefits for IPLC patients. Such approaches hold promise for enhancing the monitoring of patients with aggressive subtypes like IPLC, where early detection of recurrence is critical to improving long‐term survival outcomes.

## 4 Conclusion

This case highlights the diagnostic complexity and therapeutic nuances of HER2‐positive IPLC. The multifocal nature of IPLC, which is often challenging to detect with conventional imaging, underscores the importance of MRI for accurate staging and surgical planning. HER2‐targeted therapies offer promise in improving outcomes for patients with this aggressive subtype, but further studies are needed to establish the efficacy of these therapies specifically in IPLC. Future research should also explore the molecular drivers of HER2 positivity in IPLC, evaluate the role of AR inhibition, and investigate the potential of liquid biopsy techniques like ctDNA for monitoring disease progression. Collaborative, multicenter studies will be essential in developing standardized management protocols for IPLC and addressing the unmet clinical needs of patients with this rare and aggressive form of BC.

## Author Contributions

Conceptualization: Jiaqi Liu. Methodology: Xiaoyu Sun and Jiaqi Liu. Software and data curation: Xiaoyu Sun, Mei Wu and Jiaqi Liu. Investigation, validation, formal analysis and supervision: Jiaqi Liu. Funding acquisition, visualization and project administration: Jiaqi Liu. Resources: Xiaoyu Sun and Jiaqi Liu. Writing – original draft: Yanze Liu. Writing – review and editing: Yanze Liu and Jiaqi Liu. All authors had full access to the data, contributed to the study, approved the final version for publication, and take responsibility for its accuracy and integrity.

## Ethics Statement

We have clarified in the manuscript that the study was approved by Ethics Committee of Zibo Central Hospital (ethical approval number: 2025 Research No. 40), and informed consent was obtained from the patient for the publication of the case details.

## Consent

We had previously obtained the patient's consent for publication and there is no personal information regarding the patient in this case report.

## Conflicts of Interest

The authors declare no conflicts of interest.

## Data Availability

The datasets analyzed during the current study are available from the corresponding author on reasonable request. The transparency in data reporting in accordance with journal requirements. Corresponding author's E‐mail: 86909749@qq.com.
